# Degeneration of Axotomized Projection Neurons in the Rat dLGN: Temporal Progression of Events and Their Mitigation by a Single Administration of FGF2

**DOI:** 10.1371/journal.pone.0046918

**Published:** 2012-11-05

**Authors:** Michael L. Hendrickson, Changying Ling, Ronald E. Kalil

**Affiliations:** 1 W.M. Keck Laboratory for Biological Imaging, School of Medicine and Public Health, University of Wisconsin-Madison, Madison, Wisconsin, United States of America; 2 Department of Surgery, School of Medicine and Public Health, University of Wisconsin-Madison, Madison, Wisconsin, United States of America; 3 Department of Ophthalmology and Visual Sciences, School of Medicine and Public Health, University of Wisconsin-Madison, Madison, Wisconsin, United States of America; University of Edinburgh, United Kingdom

## Abstract

Removal of visual cortex in the rat axotomizes projection neurons in the dorsal lateral geniculate nucleus (dLGN), leading to cytological and structural changes and apoptosis. Biotinylated dextran amine was injected into the visual cortex to label dLGN projection neurons retrogradely prior to removing the cortex in order to quantify the changes in the dendritic morphology of these neurons that precede cell death. At 12 hours after axotomy we observed a loss of appendages and the formation of varicosities in the dendrites of projection neurons. During the next 7 days, the total number of dendrites and the cross-sectional areas of the dendritic arbors of projection neurons declined to about 40% and 20% of normal, respectively. The response of dLGN projection neurons to axotomy was asynchronous, but the sequence of structural changes in individual neurons was similar; namely, disruption of dendrites began within hours followed by cell soma atrophy and nuclear condensation that commenced after the loss of secondary dendrites had occurred. However, a single administration of fibroblast growth factor-2 (FGF2), which mitigates injury-induced neuronal cell death in the dLGN when given at the time of axotomy, markedly reduced the dendritic degeneration of projection neurons. At 3 and 7 days after axotomy the number of surviving dendrites of dLGN projection neurons in FGF-2 treated rats was approximately 50% greater than in untreated rats, and the cross-sectional areas of dendritic arbors were approximately 60% and 50% larger. Caspase-3 activity in axotomized dLGN projection neurons was determined by immunostaining for fractin (fractin-IR), an actin cleavage product produced exclusively by activated caspase-3. Fractin-IR was seen in some dLGN projection neurons at 36 hours survival, and it increased slightly by 3 days. A marked increase in reactivity was seen by 7 days, with the entire dLGN filled with dense fractin-IR in neuronal cell somas and dendrites.

## Introduction

Dendritic alterations, such as the formation of focal swellings, the appearance of varicosities, and the loss of distal segments, are found in many neurons in the CNS following various injuries [1]–[8]. Many of these alterations can be reversed after termination of a brief injury [9]–[12], and some neurons with partially degenerated dendrites may survive [5]. Clearly, the specific effects of an injury on the integrity of a neuron's dendritic arbor depend upon the nature of the injury and the type of neuron involved. Nevertheless, the available evidence suggests that dendritic degeneration is a common early response of neurons to injury, occurring before a commitment to cell death is made. In many injured neurons, the initial degeneration of dendrites may not be lethal, but when allowed to continue, the progressive loss of dendrites and concomitant synaptic input may lead to neuronal death. Thus, the administration of molecules that can retard early dendritic degeneration may play a role in protecting injured neurons from dying.

When the brain is injured traumatically, neurons that project to the site of injury commonly are disconnected from their synaptic targets. This disconnection, or axotomy, typically initiates a cascade of molecular and cellular events leading, in many cases, to the death of the disconnected cells [13]–[16]. However, the death of axotomized neurons often is delayed for several days after an injury, allowing for the possibility that an appropriate intervention might mitigate or prevent it.

The projection neurons in the dorsal lateral geniculate nucleus (dLGN) of the adult rat offer an excellent *in vivo* model for studying the response of neurons in the central nervous system to axonal injury. Projection neurons in the rat dLGN send their axons to the visual cortex, and, therefore, these neurons can be axotomized by removing the visual cortex [17]–[25]. When the visual cortex, principally area 17 and marginally areas 18 and 18a [26]–[28], is removed, more than 95% of the neurons in the ipsilateral dLGN persist for 3 days, but by 7 days 66% of the neurons in the dLGN have died [22], [29].

At 3 days after axotomy, cytological and biochemical changes are seen in the cell somas of many axotomized neurons; e.g., atrophy, nuclear condensation and DNA fragmentation, and the upregulation of caspase-3 activity [22], [30], [31] mark the onset of a period of rapid neuronal death that will occur during the next 3–4 days. During this time most of the axotomized dLGN projection neurons will die [22]. However, it remains unclear whether axotomized dLGN projection neurons undergo structural changes during the first three days after axotomy. In addition, it is not known at present whether any projection neurons persist in the dLGN when the total number of dLGN neurons has been reduced to approximately 33% of normal at 7 days survival. To clarify these issues, we used a modified biotinylated dextran amine (BDA) retrograde labeling method that we have developed [32] to identify dLGN projection neurons in the normal adult rat in order to reveal their structure in detail [33]. Seventy-two hours after labeling projection neurons with BDA, we axotomized these neurons by removing the visual cortex, and then studied the cytological changes that occurred in projection neurons at various intervals, ranging from 6 hours to 7 days after axotomy.

The earliest structural changes are observed in the dendrites of axotomized dLGN projection neurons at 12 hours after a lesion of visual cortex. In contrast, cell somas of axotomized projection neurons do not display obvious atrophy until 3 days after a cortical lesion, at which time many axotomized dLGN projection neurons have lost over 50% of their dendrites. During the next four days, massive cell death in the dLGN ensues, with many injured dLGN projection neurons displaying cytoplasmic vacuoles, disrupted membranes and nuclear condensation.

Many trophic factors, such as nerve growth factor (NGF), fibroblast growth factor-2 (FGF2), brain-derived neurotrophic factor (BDNF), ciliary neurotrophic factor (CNTF) and glial cell-derived neurotrophic factor (GDNF), have been demonstrated to mitigate the severity of neuronal loss after injury or disease [34]–[39]. Some of these factors have been used to mitigate axotomy-induced cell death in the rat dLGN, and among them CNTF and FGF2 have been shown to be effective [40]. A single administration of FGF2 at the time of axotomy increased the number of surviving dLGN neurons 3 months after axotomy up to 110% compared to controls [40].

FGF2, a member of a family of proteins that bind heparin and heparan sulfate, is involved in a large number of biological activities and plays a crucial role in the maintenance, survival and selective vulnerability of various neuronal populations in the normal, injured, or diseased brain [37], [41]–[44]. An injury in the CNS may trigger FGF2 gene expression and promote reactive astrocytes and injured neurons to synthesize increased amounts of FGF2, which in turn protects injured neurons from death and stimulates neuronal plasticity and tissue repair [45]. These known trophic functions of FGF2 indicate that its administration soon after a neuronal injury may prevent or mitigate the early stages of neuronal degeneration and contribute to the survival or slow the death of injured neurons.

Cell soma atrophy, the condensation of nuclear chromatin, and subsequent DNA fragmentation are generally believed to indicate that an injured neuron is undergoing apoptotic cell death [46]–[48]. Neurons undergoing apoptosis also can be distinguished by the activation of cysteine proteases, commonly known as caspases [49],[50]. Typically, these proteases exist as inactive pro-caspases in healthy cells, but under various pathological conditions, pro-caspases are cleaved, and the resulting active caspases trigger a cascade of events that leads to apoptotic cell death. Thus, caspase activation is thought to play a central role in apoptosis. In the caspase family, caspase-3 is one of the major members implicated in neurons undergoing apoptotic cell death. Currently, caspase-3 activation has been observed in neurons after various forms of injury and is considered to be a key mediator of apoptosis [51], [52].

We investigated caspase-3 activity in axotomized dLGN projection neurons immediately following injury using an antibody raised against fractin, a 32 kDa N-terminal actin fragment that results exclusively from the cleavage of beta actin by activated caspase-3 [53]–[56]. Thus while other proteases such as calpain may be involved in the death of axotomized dLGN neurons, the presence of fractin confirms the involvement of caspase-3. The present results demonstrate that the majority of axotomized dLGN projection neurons undergo apoptotic cell death, and they provide details about the initiation, duration, and cytological distribution of activated caspase-3 activity in injured dLGN projection neurons.

## Materials and Methods

### Ethics Statement

#### Research Animals

All animal handling and procedures were performed in accordance with protocols for these studies that have been approved by the Institutional Animal Care and Use Committee at the University of Wisconsin-Madison. All surgery was performed aseptically under deep anesthesia, and every attempt was made to minimize pain and discomfort.

### Research Animals

#### Overview

Sixty-three adult male Holtzman rats (250–275 grams) were used in this study. Forty-eight rats received bilateral injections of biotinylated dextran amine (BDA) into the visual cortex and five rats received injections of Texas Red conjugated dextran (TRD) into the right visual cortex. Three days after making the BDA or TRD injections, all of the injected rats, as well as 10 additional, uninjected animals, received a unilateral lesion of the right visual cortex. Twelve animals that received both bilateral injections of BDA and a lesion of the visual cortex also received a single administration of FGF2 into the lesion cavity (see below). The rats then were allowed to survive for periods ranging from 6 hours to 7 days before being deeply anesthetized and perfused through the heart with 4% paraformaldehyde.

#### BDA-labeling of dLGN Projection Neurons

**Figure 1:**
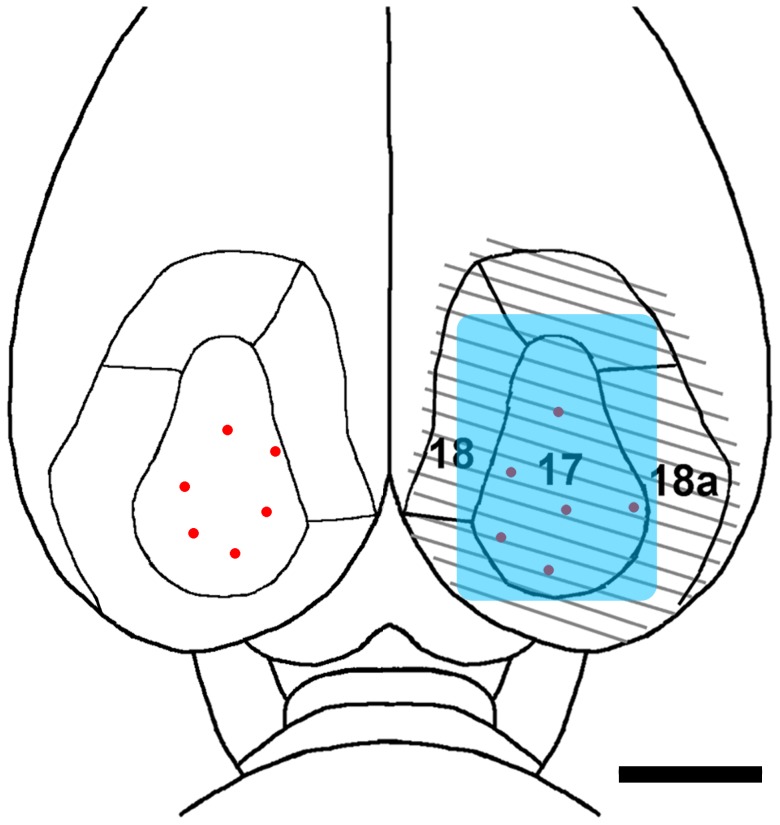
A schematic diagram representing a dorsal view of the caudal portion of the rat brain, indicating the striate (area 17) and extrastriate (areas 18a and 18) visual cortex. The lesion site is indicated by the striped area and each BDA or TRD injection is represented with a red dot. The blue rectangle indicates the placement of the gelatin sponge saturated with FGF2. Scale bar: 3.0 mm.

Animals were anesthetized initially with an intramuscular injection of a mixture of 70 mg/kg of ketamine HCl and 7 mg/kg of xylazine. After being placed in a stereotaxic head holder, anesthesia was maintained with 1–2% isoflurane. Under aseptic conditions, bone flaps were cut bilaterally to expose the dorsal surface of the visual cortex, cortical areas 17, 18, and 18a (Figure 1) as defined [26]–[28]. A 5% w/v solution of BDA (3,000 MW, Invitrogen) in glass distilled water was pressure-injected bilaterally into the visual cortex through a glass pipette with a tip diameter of approximately 10 µm. Typically, five BDA injections, each 0.1 µL, were made slowly over 10 minutes at a depth of 0.8 mm below the surface of the brain in the approximate center of layer IV in cortical area 17 (Figure 1). When each injection was complete, the pipette was left in place for an additional 2 minutes before withdrawing it slowly to reduce back-flowing of the injected solution. The bone flaps then were replaced and the scalp incision was closed with wound clips. The rats were returned to their home cages after recovery on a warming pad.

#### Injection of Texas Red Conjugated Dextran (TRD)

Animals were anaesthetized as described and placed in a stereotaxic head holder. Under aseptic conditions, a bone flap was cut over the dorsal surface of the right visual cortex to expose cortical areas 17, 18, and 18a (Figure 1). Five 0.1 µL injections of a 5% w/v solution of TRD (3,000 MW, Invitrogen) were made into the visual cortex following the same procedures described above for BDA injections. Upon completion of the injections, the animals were allowed to recover as mentioned.

#### Unilateral Removal of the Visual Cortex and Administration of FGF2

Three days after the injection of BDA or TRD, the animals were anesthetized again. The wound clips placed previously were detached, and the bone flap above the right visual cortex was removed to re-expose the visual cortex, cortical areas 17, 18 and 18a (Figure 1), which was then removed by subpial suction. The lesion cavity was filled with a gelfoam sponge (4.2×6.0×1.5 mm) moistened in 0.9% saline, the bone flap was replaced, and the scalp incision was closed with wound clips. Some animals (n = 12) received a gelfoam sponge that was saturated with FGF2 (0.26 mg/mL) for a total dose of approximately 40–45 µL or 10 µg per lesion site. After recovery from anesthesia on a warming pad, the animals were returned to their home cages.

#### BDA-Injected Animals: Tissue Preparation and Cytochemistry

The FGF2 treated rats and untreated rats were allowed to survive for 6, 12, 24, 48, 72 hours, or 7 days after the lesion of visual cortex (n = 6 for each group), and then they were deeply anesthetized with a mixture of 135 mg/kg of ketamine HCl and 14 mg/kg of xylazine and perfused transcardially with 0.9% NaCl followed by 4% paraformaldehyde in 0.1 M phosphate buffer (pH 7.4). The brains were post-fixed overnight in 4% paraformaldehyde and then sectioned coronally at 75 µm with a vibrating blade microtome (Leica, VT1000s). Sections through the dLGN were collected and washed thoroughly in 0.1 M phosphate buffer. Retrogradely transported BDA was detected by using an avidin-biotin-peroxidase complex (Vector Lab, Elite ABC Kit) and visualized using diaminobenzidine (DAB) as a chromogen. Finally, the sections were processed for gold/silver intensification and prepared for examination under brightfield microscopy [32].

#### Caspase-3: Tissue Preparation and Immunocytochemistry

Fifteen rats were used for the evaluation of caspase-3 activity. The animals survived for 24, 36, 48, 72 hours or 7 days after the visual cortex had been removed (n = 3 each group), and then were anesthetized, perfused with 4% paraformaldehyde, and their brains were sectioned at 40 µm as described above. Caspase-3 activity was detected by using a rabbit polyclonal antibody (a gift from Dr. Greg Cole of UCLA) that had been raised against the C-terminus of a 32-kDa N-terminal actin fragment, fractin, which is generated specifically by caspase-3 cleavage [53]–[56]. The brain sections first were incubated in blocking solution (0.1 M PBS containing 5% normal goat serum and 2.5% bovine serum albumin) for 1 hour at room temperature (RT), followed by a 90 minute incubation at RT in the primary antibody diluted 1∶400 in blocking solution. The sections then were rinsed thoroughly and exposed to a FITC conjugated or a biotinylated secondary antibody for 90 minutes at RT. The sections incubated with the biotinylated secondary antibody were further incubated with an avidin-biotin-peroxidase complex (Vector, Elite ABC Kit) and reacted with DAB. The DAB-reacted sections were studied under a light microscope at magnifications ranging from 200X to 1000X to examine fractin immunoreactive (fractin-IR) products. Control sections were processed identically, but the primary antibody was omitted. Sections containing fluorescently-labeled cells (TRD-labeled dLGN neurons and FITC-labeled fractin) were studied with a confocal microscope (Bio-Rad Radiance 2100 MP Rainbow).

### Statistical Analysis

#### BDA-Labeled Neurons

All BDA-labeled neurons were examined with a brightfield microscope at a magnification of 1000X. Since the BDA labeling quality varied significantly among individual dLGN projection neurons, structural analysis was restricted to projection neurons that appeared to be completely labeled. Using the same criteria as described previously [33], the cell soma and dendrites of a neuron considered to be completely labeled with BDA and contained within a single 75 µm section on a clean background without interference from other labeled neuronal profiles were analyzed. Observing these constraints stringently, completely labeled projection neurons in the dLGN were traced with the aid of a camera lucida drawing tube attachment. The number of dendrites was counted, and the dendritic arbors were drawn. In addition, labeled cell somas that were clearly delineated were traced for cell soma size analysis. The tracings and drawings then were digitized and the area enclosed by each was computed as previously described [33]. BDA-labeled projection neurons in each of the three classes previously identified, radial, basket and bipolar [33], were grouped collectively for analysis according to survival time and FGF2 treatment. The group means of the cross-sectional areas of neuronal somas and dendritic arbors, and of the number of dendrites per cell ± the standard error of the mean were calculated. BDA-labeled projection neurons in the dLGN contralateral to the lesion of the visual cortex were equivalent in structure to labeled dLGN projection neurons in normal controls at each time point studied. Therefore, we analyzed the labeled contralateral neurons quantitatively and used the cell soma and dendritic arbor size results as control data for comparison with BDA-labeled projection neurons in the dLGN ipsilateral to the cortical lesion. Differences between the groups were evaluated statistically with an unpaired Student's two-tailed *t*-test. Differences between means were considered significant if they exceeded p<0.01.

#### Neurons Stained for Fractin

At 36, 48 and 72 hour survival times, it was possible to distinguish individual fractin-IR neurons in the dLGN ipsilateral to a lesion of the visual cortex. At each of these survival times the number of fractin-IR neurons in the dLGN was counted. The group means ± the standard error of the mean were calculated, and differences between group means were evaluated statistically with an unpaired Student's two tailed *t*-test. Differences between means were considered significant if p<0.01.

## Results

### Sequence of Structural Changes in Axotomized dLGN Projection Neurons

**Figure 2:**
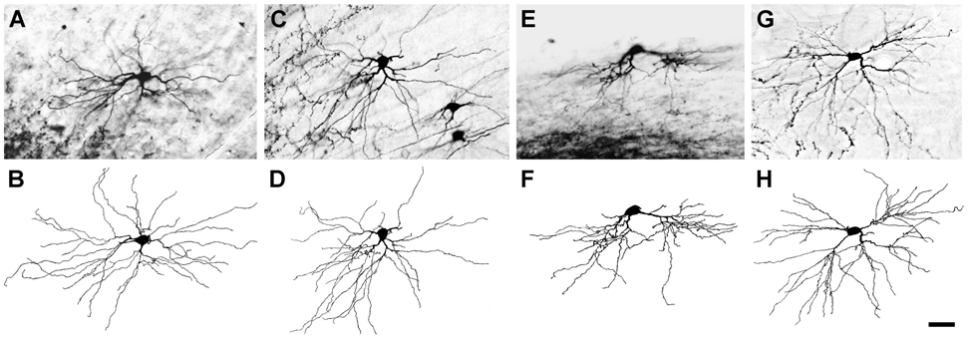
dLGN projection neurons labeled by injections of BDA into the visual cortex. The micrograph of each cell is accompanied by a camera lucida drawing of the cell to illustrate all labeled dendrites, particularly those that are out of focus in the plane of the micrograph. (**A**, **B**) A radial cell from an unlesioned rat with dendrites arrayed around the cell soma. (**C**, **D**) A basket cell from an unlesioned rat with dendrites distributed asymmetrically. (**E**, **F**) A bipolar cell from an unlesioned rat with dendrites emerging from two poles of the cell soma spaced approximately 180° apart. (**G**, **H**) A radial cell from a rat that received a lesion of the contralateral visual cortex 48 hours prior. Images adapted in part from [32]. Scale bar: 50 µm.

The structure of dLGN projection neurons retrogradely labeled with BDA in normal rats has been described in detail in a previous report [33]. This report demonstrated that BDA-labeled projection neurons in the dLGN can be grouped into three broad classes of neurons: radial, basket, or bipolar cells, based on the distribution of dendrites around the cell soma (Figures 2A–F). In animals that received a unilateral lesion of the visual cortex and survived from 6 hours to 7 days, BDA-labeled projection neurons located in the dLGN contralateral to the lesion were indistinguishable from dLGN projection neurons seen in normal animals (Figures 2G, H). However, in the dLGN ipsilateral to the cortical lesion, labeled projection neurons displayed significantly altered morphology at each survival period studied, with the exception of 6 hours survival when no structural changes were evident. The structural changes observed at each of the later survival periods are described below.

#### 12 Hours

**Figure 3:**
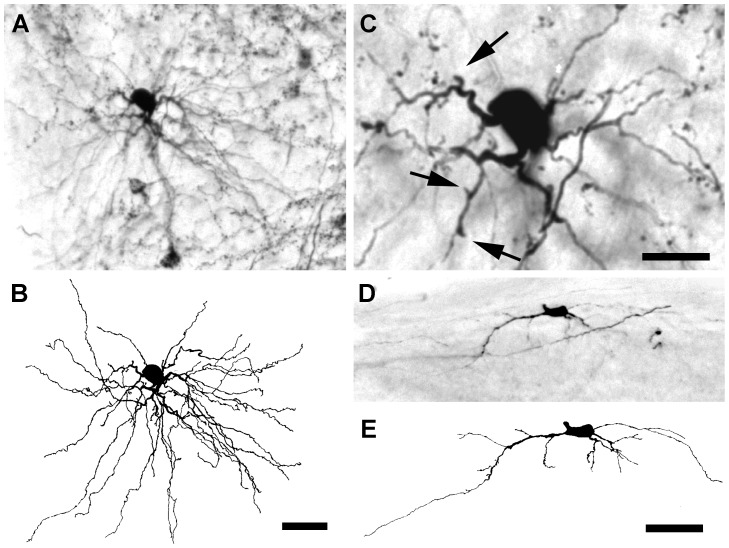
Response to axotomy from a lesion of the visual cortex at 12 hours. (**A**) Micrograph of a BDA-labeled basket cell in the dLGN ipsilateral to the lesion with nearly normal dendrites. (**B**) A camera lucida drawing of the neuron shown in **A**. (**C**) A higher magnification micrograph of the basket cell in **A** showing dendritic appendages (arrows). (**D**) A BDA-labeled bipolar cell with severely degenerated dendrites. (**E**) A camera lucida drawing of the bipolar cell shown in **D**. Scale bars: 50 µm (**A**, **B**, **D**, **E**) and 20 µm (**C**).

Twelve hours after axotomy, cytological changes of various degrees were evident in some BDA-labeled projection neurons, although some axotomized neurons, principally radial cells, were morphologically similar to control neurons with normal-sized cell somas that supported many secondary dendrites decorated with appendages. While many radial cells displayed relatively minor structural changes at 12 hours survival, basket cells (Figures 3A, B) often exhibited dendrites that appeared to support truncated branches (arrows in Figure 3C). Bipolar cells, however, showed more severe dendritic alterations than radial or basket cells. Injured bipolar cells retained 1–2 long, thin dendrites that frequently were beaded distally and lacked appendages. Other dendrites lacked side branches, were truncated in length, and appeared swollen (Figures 3D, E). Thus in contrast to radial or basket cells, which showed relatively minor structural alterations at 12 hours survival, 3 out of 10 projection neurons with clear dendritic degeneration were bipolar cells, a ratio three times greater than the number of bipolar cells to all dLGN projection neurons in control animals [33].

#### 48 Hours

**Figure 4:**
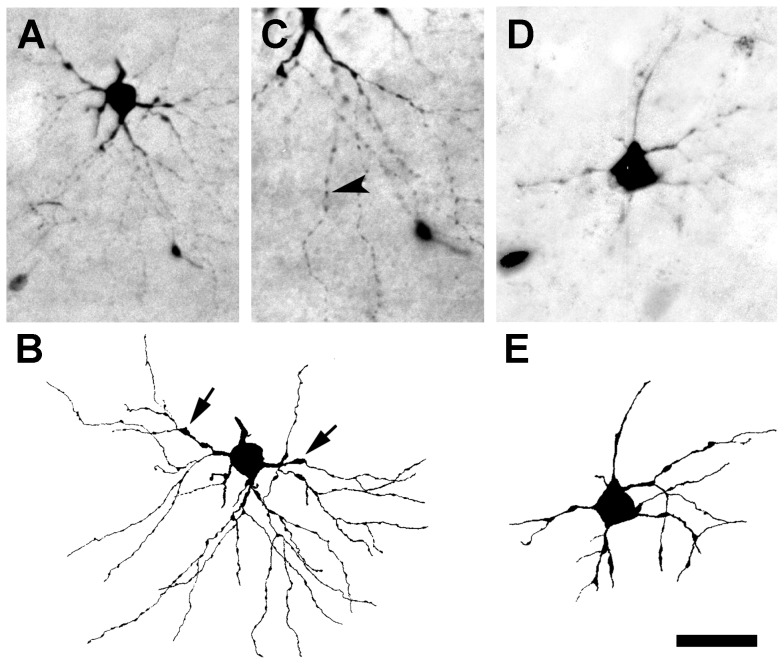
Response to axotomy from a lesion of the visual cortex at 48 hours. (**A**) Micrograph of a BDA-labeled basket cell with slender, beaded dendrites 48 hours after axotomy. (**B**) A camera lucida drawing of the neuron shown in **A**. Arrows indicate swollen primary dendrites, and beaded dendrites are evident. (**C**) At higher magnification, the detailed morphology of the beaded dendrites (arrowhead) of the cell shown in **A** can be visualized. (**D**) A degenerated radial cell showing the loss of primary and secondary dendrites. (**E**) A camera lucida drawing of the radial cell shown in **D**. Scale bar: 50 µm (**A**, **B**, **D**, **E**) and 25 µm (**C**).

Within the next 36 hours, the number of BDA-labeled projection neurons with degenerated dendrites increased steadily, while the number of labeled projection neurons with nearly normal dendrites gradually disappeared. The majority of BDA-labeled neurons lost many of their dendritic branches and distal segments during this period, but their cell somas, primary and some secondary dendrites appeared relatively normal. However, labeled projection neurons with multiple long dendrites were occasionally seen (Figure 4A), but their primary dendrites were frequently swollen (arrows in Figure 4B), and their secondary dendrites always had a beaded appearance and lacked appendages (arrowhead in Figure 4C). Occasionally, severely degenerated cells were observed that displayed a marked loss of primary and secondary dendrites (Figures 4D, E).

#### 72 Hours

**Figure 5:**
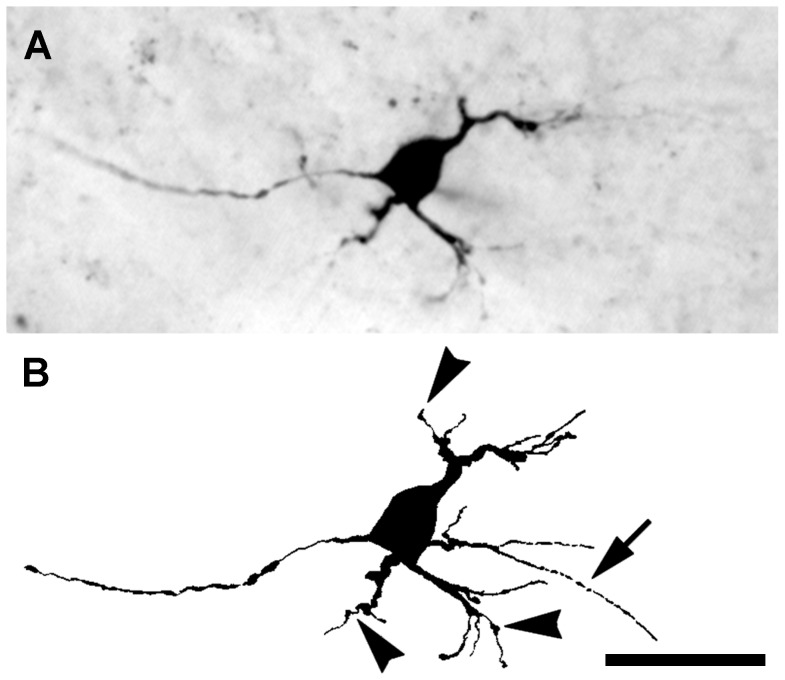
Response to axotomy from a lesion of the visual cortex at 72 hours. (**A**) A micrograph of a BDA-labeled dLGN projection neuron with swollen primary dendrites and only a few short remnants of secondary dendrites remaining. (**B**) A camera lucida drawing of the neuron shown in **A**. The arrow indicates a discontinuous distal segment of a dendrite. Arrowheads indicate bulb-like protrusions on dendrites. Scale bar: 50 µm.

Seventy-two hours after a lesion of the visual cortex, a typical BDA-labeled dLGN projection neuron had a relatively small cell soma, 125–150 µm^2^, and none of the remaining dendrites was longer than 150 µm. In these projection neurons, some primary dendrites remained, but only short remnants of secondary dendrites were evident (Figures 5A, B). The remaining dendrites were varicose throughout their length and often appeared segmented distally (arrow in Figure 5B). Truncated dendrites with bulbous protrusions but lacking dendritic appendages were observed frequently (arrowheads in Figure 5B). Occasionally, cell membrane blebbing was observed in projections neurons at 72 hours after axotomy.

#### 7 Days

**Figure 6:**
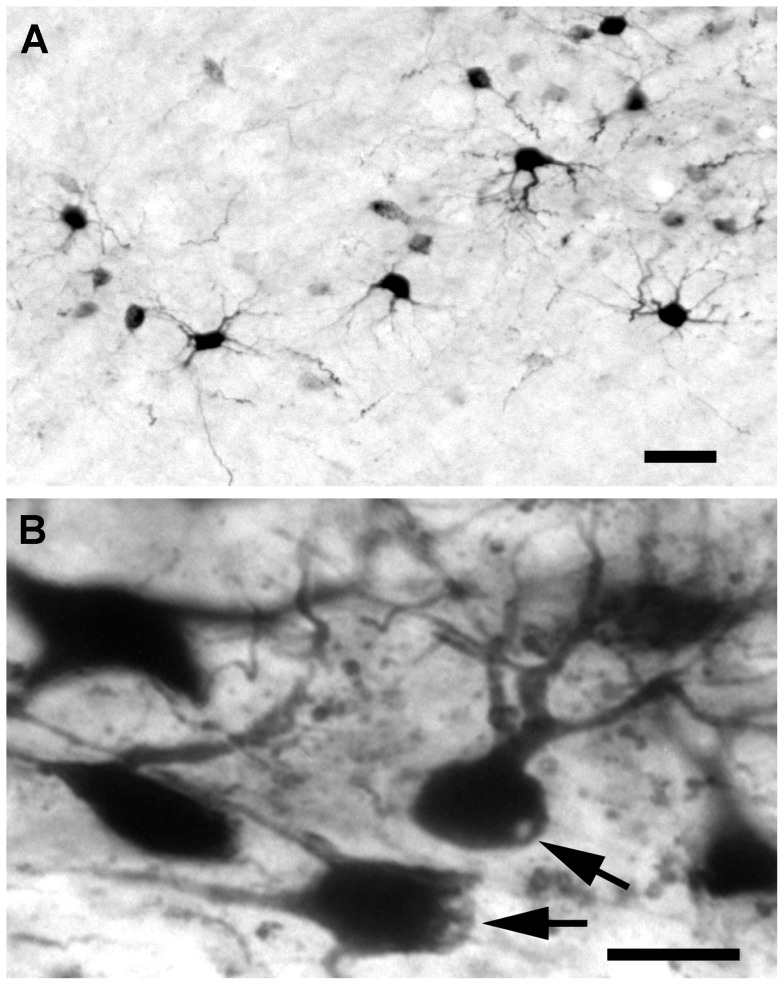
Response to axotomy from a lesion of the visual cortex at 7 days. (**A**) The majority of these neurons have multiple primary dendrites, few short secondary dendrites, and appear to be degenerated radial cells. (**B**) The cell somas of many dLGN projection neurons appear to contain vacuoles (upper arrow) and also display membrane blebbing (lower arrow). Scale bar: 50 µm (**A**) and 20 µm (**B**).

In rats that survived 7 days after a lesion of the visual cortex, a low density of BDA-labeled projection neurons was observed in the dLGN ipsilateral to the cortical lesion. These neurons uniformly displayed fewer and shorter dendrites than those of labeled control projection neurons in the contralateral dLGN (Figure 6A). Vacuoles and membrane blebbing could be seen in the cytoplasm of the cell somas of some surviving axotomized projection neurons (Figure 6B), indicating near end-stage degeneration. The primary dendrites of many surviving projection neurons were arranged evenly around cell somas, indicating that these neurons were likely radial cells. To a lesser extent, basket cells also remained, but only a small number of bipolar cells was evident. BDA-labeled oval profiles resembling small cell somas, less than 100 µm^2^, that lacked dendrites also were seen. These BDA-labeled profiles presumably reflected the cell somas of end-stage dying or dead projection neurons prior to being removed from the dLGN by phagocytes. While these results provide direct evidence that some projection neurons in the dLGN ipsilateral to a lesion of the visual cortex survive for at least 7 days after a lesion, none of the observed surviving projection neurons appeared normal, which is perhaps to be expected as the total number of neurons in the ipsilateral dLGN has been reduced by two thirds at this survival time [22].

### Axotomy-Induced Dendritic Degeneration of dLGN Projection Neurons Is Directly Related to Postoperative Survival Time

**Figure 7:**
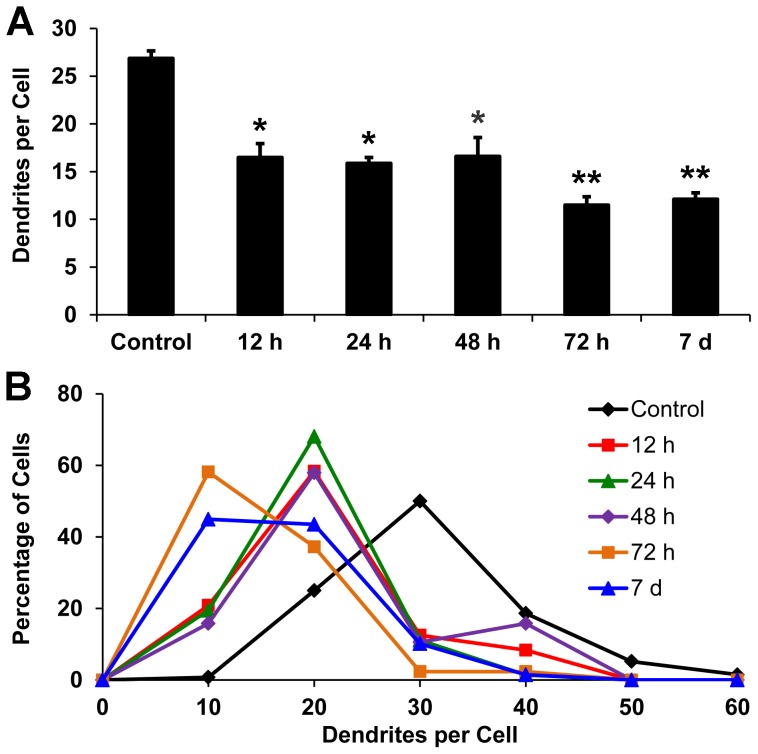
The mean number of dendrites of dLGN projection neurons contralateral (Control) and ipsilateral to a lesion of the visual cortex in rats that survived for intervals ranging from 12 hours to 7 days following the lesion. (**A**) The mean number of dendrites at all intervals studied is significantly different from controls, *p<0.01 (two-tailed *t*-test; n = 6 in each group). Moreover, the mean number of dendrites in rats surviving for 72 hours or 7 days differs significantly from that in rats surviving for 12 hours, **p<0.01 (two-tailed *t*-test, n = 6 in each group). (**B**) Frequency distributions of the number of dendrites per projection at each survival period studied. Note that a shift in the frequency distribution in the number of dendrites per dLGN projection neuron is evident as early as 12 hours after a lesion of the visual cortex.

**Figure 8:**
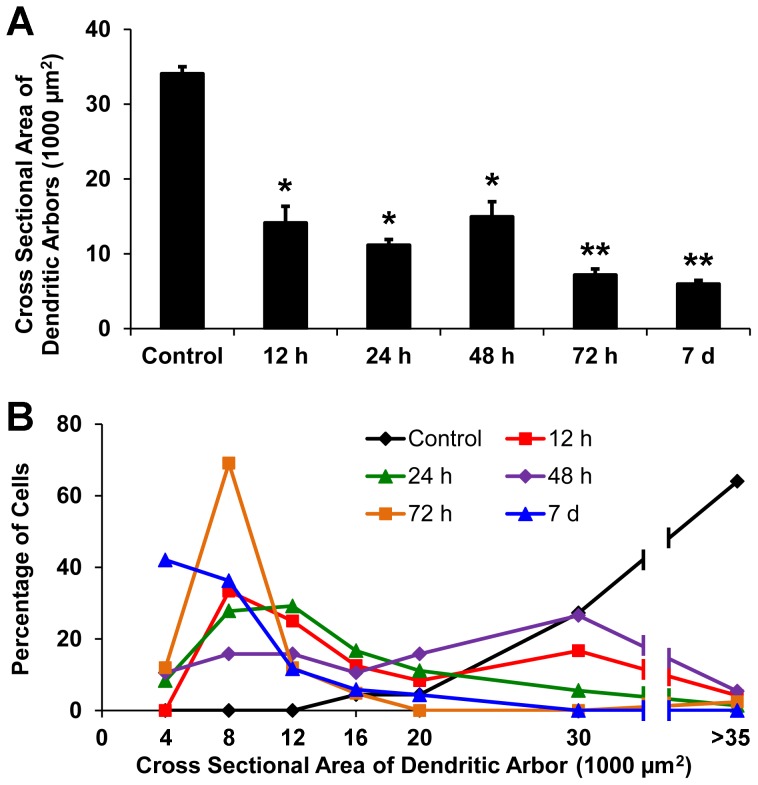
The mean cross-sectional area of dendritic arbors of dLGN projection neurons contralateral (Control) and ipsilateral to a lesion of the visual cortex in rats that survived for intervals ranging from 12 hours to 7 days following the lesion. (**A**) The mean cross-sectional area at all intervals studied is significantly different from controls, *p<0.01 (two-tailed *t*-test; n = 6 for each group). Moreover, the mean cross-sectional area in rats surviving for 72 hours or 7 days differs significantly from that in rats surviving for 12 hours, **p<0.01 (two-tailed *t*-test, n = 6 for each group). (**B**) Frequency distributions of the cross-sectional area of the dendritic arbors at each survival period studied. Note that approximately 90% of the BDA-labeled cells in control animals have large dendritic arbors with cross-sectional areas of 30×10^3^ µm^2^ or larger, while at 12 hours survival only about 20% of the labeled cells retained dendritic arbors of this size. At 72 hours and 7 days survival, the cross-sectional area of the dendritic arbors of the majority of labeled dLGN projection neurons was 8×10^3^ µm^2^ or smaller.

In order to characterize sequential structural changes in axotomized dLGN neurons, we investigated changes in the number of primary dendrites, the total number of dendrites per cell, and the cross-sectional area of dendritic arbors at various intervals after axotomy. In general, the total number of dendrites per cell and the cross-sectional area of dendritic arbors were reduced as postoperative survival time increased (Table 1). The number of dendrites per cell was reduced to about 60% of normal at 12 hours survival, declined further to 43% of normal at 72 hours survival, and then plateaued for the next 4 days (Figure 7A). Similarly, the frequency distribution of the number of dendrites per cell shifted to smaller values at 12 and 72 hours survival (Figure 7B). The first shift was caused by a significant reduction in the number of projection neurons containing more than 20 dendrites, and the second shift resulted from the reduction of neurons containing more than 10 dendrites (Table 1). The cross-sectional area of the dendritic arbors showed more severe changes with increasing survival periods than the number of dendrites per cell. This is primarily due to the rapid loss of distal dendritic segments, which resulted in a decrease in the cross-sectional area of dendritic arbors to about 45% and 20% of controls at 12 and 72 hours survival, respectively (Figure 8A). Similarly, the frequency distribution of the cross-sectional area of dendritic arbors was shifted to significantly smaller values at 72 hours survival (Figure 8B).

**Table 1:** Cell Class-Related Differences in Vulnerability to Axotomy.

	Cell Class	C	12 h	24 h	48 h	72 h	7 d
Total Population (%)	Radial	55	52	57	70	63	69
	Basket	35	32	29	20	30	28
	Bipolar	10	16	14	10	7	3
Dendritic Arbors >20,000 µm in Cross-Sectional Area (%)	Radial	100	31	2	25	4	0
	Basket	100	13	5	50	0	0
	Bipolar	100	0	0	0	0	0
Dendritic Arbors >10,000 µm in Cross-Sectional Area (%)	Radial	100	93	59	63	18	12
	Basket	100	51	43	100	8	5
	Bipolar	100	100	33	0	0	0
Cells with >20 Dendrites (%)	Radial	100	38	26	25	7	20
	Basket	98	0	16	50	0	5
	Bipolar	58	0	10	0	0	0
Cells with >10 Dendrites (%)	Radial	100	85	95	88	57	69
	Basket	100	100	83	100	92	45
	Bipolar	100	88	76	50	33	50

### Cell Class-Related Differences in the Response of dLGN Projection Neurons to Axotomy

We have classified dLGN projection neurons structurally into three classes: radial, basket and bipolar cells [33]. This classification scheme is based in part on the spatial distribution and number of primary dendrites displayed by projections neurons. As primary dendrites are evident in axotomized neurons, the three classes of projection neurons can be distinguished as these neurons undergo structural changes after axotomy.

In order to determine whether there are differences among the three classes of dLGN projection neurons in response to axotomy, we analyzed the structural changes occurring within each class of neurons as a function of survival time. As shown in Table 1, during the first 24 hours after axotomy the number of neurons in each cell class as a percentage of all dLGN projection neurons remained largely unchanged. However, at 48 hours survival, the relative percentage of radial cells increased while the relative percentages of basket and bipolar cells decreased. With respect to the size of dendritic arbors, all three classes of projection neurons (n = 185) in control animals had dendritic arbors with cross-sectional areas greater than 2.0×10^4^ µm^2^. Twelve hours after axotomy, about 30% of radial cells retained dendritic arbors larger than 2.0×10^4^ µm^2^, but the percentage of basket cells with dendritic arbors greater than 2.0×10^4^ µm^2^ had fallen dramatically to 13% and no bipolar cells had dendritic arbors of this size. At 24 hours survival nearly 60% and 45% of radial and basket cells, respectively, retained dendritic arbors greater in size than 1.0×10^4^ µm^2^, but only 33% of the surviving bipolar cells displayed dendritic arbors greater than 1.0×10^4^ µm^2^. Comparable declines in the number of dendrites per projection neuron with increasing survival times were observed. Generally, radial cells retained more dendrites than basket cells which in turn retained more dendrites than bipolar cells. Seven days after axotomy, 69% of the surviving dLGN projection neurons were radial cells, 28% were basket cells, and at 3% of the total number of surviving projection neurons, bipolar cells had largely disappeared from the dLGN.

### dLGN Projection Neuron Cell Soma Atrophy after Axotomy

**Figure 9:**
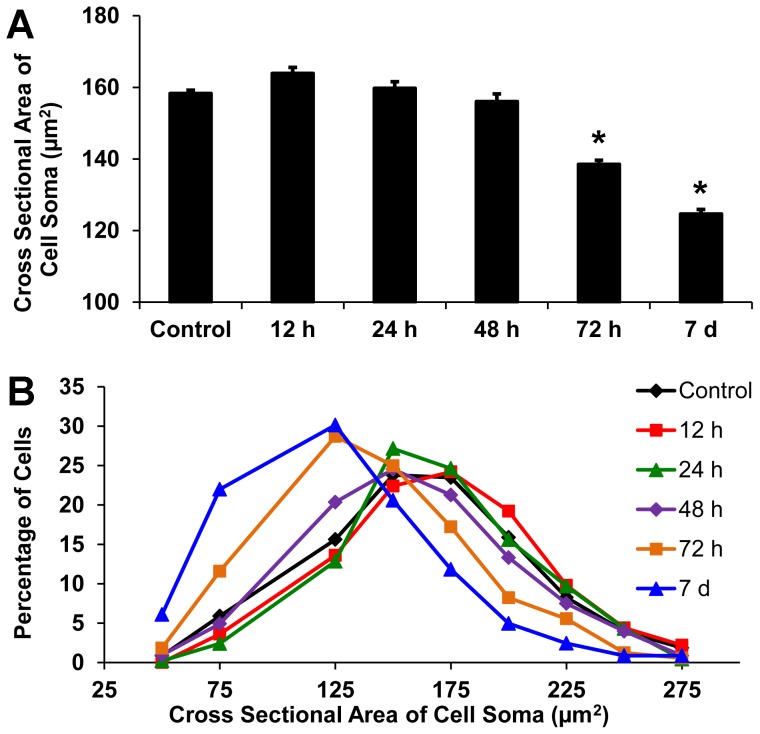
The mean cross-sectional area of the cell soma of dLGN projection neurons contralateral (Control) and ipsilateral to a lesion of the visual cortex in rats that survived for intervals ranging from 12 hours to 7 days following the lesion. (**A**) The mean cross-sectional area at 72 hours and 7 days survival is significantly different from controls, *p<0.01 (two-tailed *t*-test; n = 6 for each group). (**B**) Frequency distributions of the mean cross-sectional area of the cell soma at each survival period studied. Note the leftward shift in distributions from the 72 hour and 7 day survival periods.

In order to determine the time course of cell soma atrophy of axotomized dLGN projection neurons, the mean cross-sectional areas of over 10,000 BDA-labeled neuronal somas in the dLGN were measured at different intervals after axotomy. Mean cell soma size was similar for projection neurons in the dLGN contralateral to a lesion of the visual cortex and in the dLGN ipsilateral to a cortical lesion in animals that survived for 48 hours or less (Figure 9A). Moreover, the frequency distributions of cell soma sizes were similar for projection neurons in the dLGN contralateral to a cortical lesion and for cells ipsilateral to the lesion in animals surviving for 48 hours or less (Figure 9B). However, mean cell soma size was significantly reduced, and the frequency distributions of cell soma sizes were shifted to smaller values in the dLGN ipsilateral to a visual cortex lesion in animals that survived for 72 hours or more (Figures. 9A,B). The frequency distributions in Figs. 7, 8, and 9 are included only to complement and expand upon the data presented as histograms in these figures, and therefore have not been analyzed statistically.

### Mitigation of Dendritic Degeneration in Axotomized dLGN Projection Neurons at 72 Hours and 7 Days Survival Following a Single Administration of FGF2

**Figure 10:**
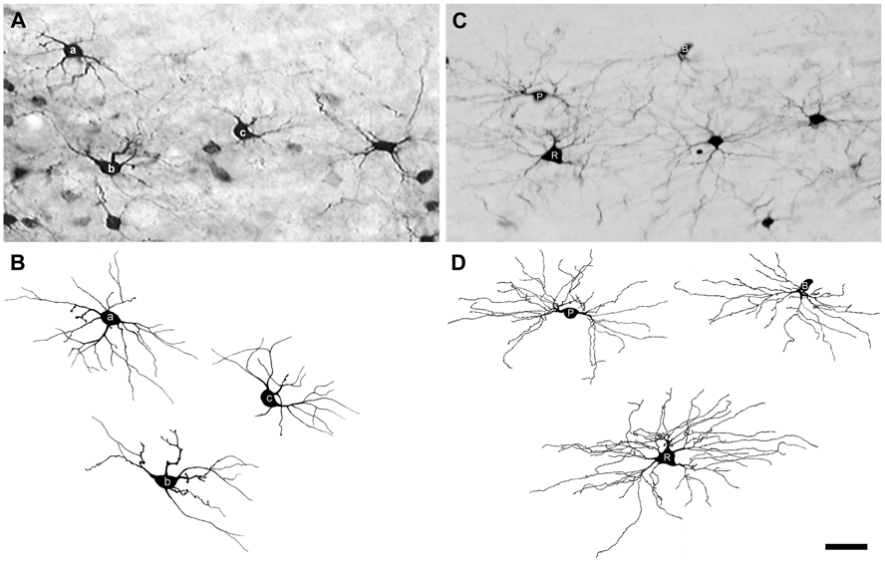
BDA-labeled dLGN projection neurons 7 days after axotomy in untreated and FGF2-treated rats. (**A**) Micrograph of dLGN projection neurons showing that the majority of surviving neurons appear to be radial or basket cells. (**B**) Camera lucida drawings of the three neurons (a, b, and c) identified in **A** illustrating their shortened dendrites. (**C**) Micrograph of dLGN projection neurons with normal appearing dendrites in an FGF2-treated rat. (**D**) Camera lucida drawings of the bipolar cell (P), basket cell (B), and radial cell (R) identified in **C**. Scale bar: 50 µm.

At 7 days after a lesion of the visual cortex, the surviving projection neurons in the ipsilateral dLGN, most of which were radial cells or basket cells, usually supported 10–12 dendrites (Figure 10A, B). In rats that were administered FGF2 at the time of the cortical lesion, dLGN projection neurons showed relatively minor dendritic changes compared to those seen in untreated animals. Some BDA-labeled axotomized dLGN neurons in FGF2-treated rats appeared similar cytologically to cells seen in normal animals (Figure 2) as late as 7 days after axotomy. Figure 10 (C, D) shows five BDA-labeled dLGN projection neurons in an FGF2-treated rat, a bipolar cell (P), a basket cell (B) and three radial cells (R), with dendrites that appear to be normal 7 days after axotomy.

**Figure 11:**
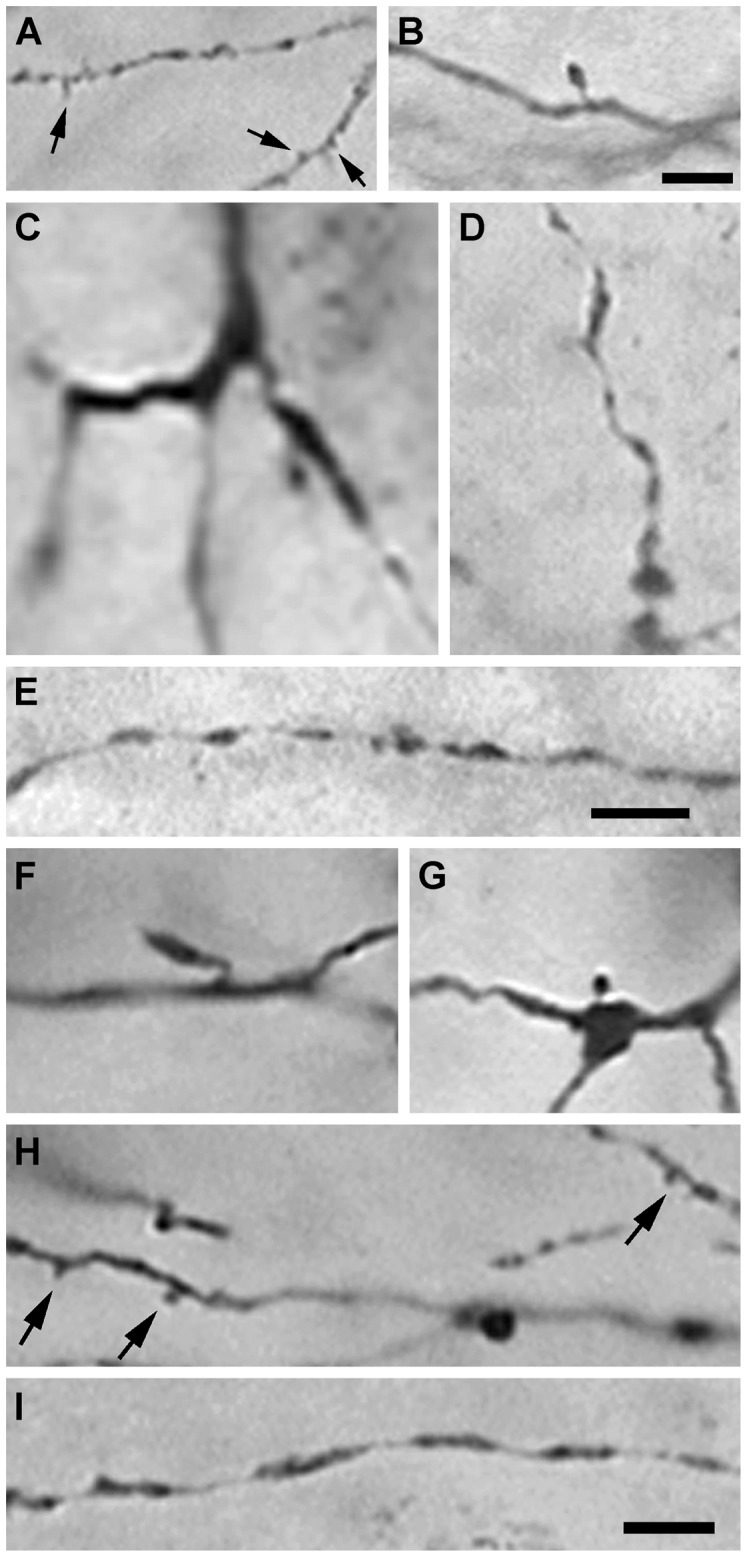
Dendritic appendages of dLGN projection neurons retrogradely labeled with BDA in normal rats, in rats that received a lesion of the visual cortex, and in rats with a lesion of visual cortex rats that were treated with FGF2. (**A**) Tuft-like appendages (arrows) and (**B**) spiny appendages from a normal rat. (**C**) Irregularly swollen proximal and (**D**) distal dendrites typically seen in dLGN projection neurons 7 days after axotomy in untreated rats. (**E**) A small caliber beaded dendrite in an untreated rat. (**F**, **G**) Spiny dendritic appendages on projection neurons 7 days after axotomy in an FGF2-treated rat. (**H**) Tuft-like dendritic appendages (arrows) and (**I**) a distal dendritic segment with a beaded appearance in an FGF2-treated rat. Scale bars: 10 µm.

**Figure 12:**
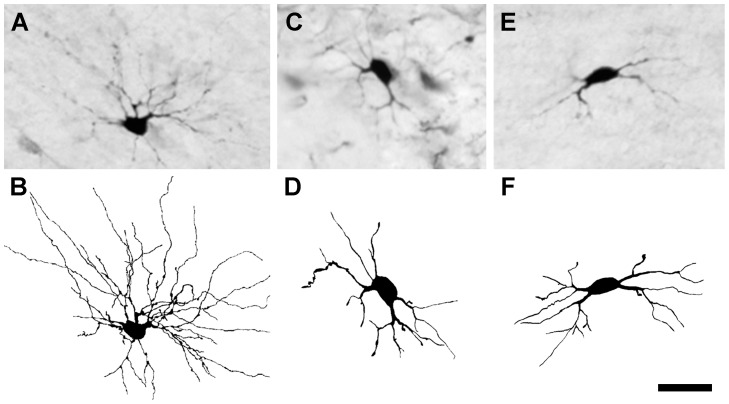
BDA-labeled dLGN projection neurons with partially degenerated dendrites 7 days after axotomy in an FGF2-treated rat. A micrograph of each cell is accompanied by a camera lucida drawing: (**A**, **B**) radial cell, (**C**, **D**) basket cell, and (**E**, **F**) bipolar cell. Scale bar: 50 µm.

Labeled cells in each of the three classes exhibited dendrites with various types of appendages: “tufted” appendages were short protuberances appearing mainly on the distal branches of dendrites (Figure 11A), “spine-like” appendages had fine stalks with a single ovoid head and were frequently seen extending from the middle segment of a dendrite (Figure 11B), and less frequently “grape-like” appendages (not illustrated) had short stalks terminating in a cluster of ovoid bulbs and emerged at the bifurcation of primary dendrites or along the proximal segments of secondary dendrites. However in animals that received a lesion of the visual cortex, the dendrites of surviving projection neurons 7 days after axotomy had relatively smooth surfaces with few appendages (Figures 11C–E). The proximal portions of the dendrites of these neurons often appeared swollen (Figure 11C), while the distal segments almost always exhibited a beaded appearance (Figures 11D, E). In rats treated with FGF2, many axotomized projection neurons supported dendrites that appeared intact and displayed appendages typical of dendrites on normal projection neurons (Figure 11F–H). However the dendrites of some axotomized neurons in FGF2-treated rats that survived for 7 days after axotomy exhibited beaded distal segments (Figure 11I), while other neurons supported dendrites with significant degeneration, not unlike those seen in untreated rats at 7 days survival. These neurons had fewer and shorter dendrites compared to those in normal rats, and often were swollen and beaded with few appendages (Figure 12).

BDA-labeled projection neurons with dendritic degeneration were distributed throughout the dLGN. Strikingly, axotomized projection neurons with severe dendritic degeneration in FGF2-treated rats often were seen in the immediate vicinity of projection neurons with dendrites that appeared almost normal. Such variability in dendritic survival is unlikely to be due to variables associated with the cortical lesion or the administration of FGF2 as adjacent projection neurons in the dLGN project to adjacent loci in the visual cortex, and, therefore, each projection neuron would have been affected similarly by the cortical lesion and the FGF2 treatment.

**Figure 13:**
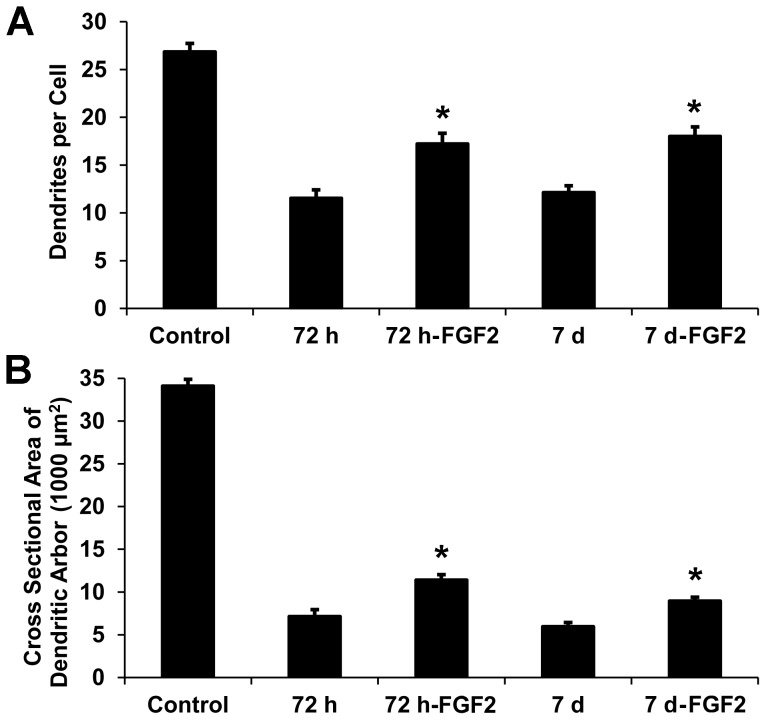
Dendritic degeneration of axotomized dLGN projection neurons in untreated rats and FGF2-treated rats at 72 hours and 7 days after a lesion of the visual cortex. At each survival period, 72 hours and 7 days, a single treatment with FGF2 lessens, but does not eliminate, a loss in the number of dendrites or a reduction in the size of the dendritic arbors of axotomized dLGN projection neurons. (**A**) The mean number of dendrites per cell is 50% greater at 72 hours and 7 days after axotomy in FGF2-treated rats compared to untreated rats. (**B**) The mean cross-sectional areas of the dendritic arbors of projection neurons are 60% and 50% larger at 72 hours and 7 days, respectively, in FGF2-treated rats compared to untreated rats. *p<0.01 for treated compared to untreated rats (two-tailed *t*-test, n = 6 for each group). Data for control, 72 hour untreated, and 7 day untreated rats are the same as appear in Figures 7A and 8A.

Quantitative analysis showed that axotomized dLGN projection neurons in FGF2-treated rats had, on average, 50% more dendrites than untreated controls at 72 hours and 7 days survival. Similarly, the dendritic arbors of axotomized dLGN projection neurons in FGF2-treated rats were 60% larger than those seen in axotomized cells in untreated rats at 72 hours survival and 50% larger at 7 days survival (Figure 13). Statistically, both the mean number of dendrites and the mean cross-sectional area of dendritic arbors of dLGN projection neurons were significantly greater in FGF2-treated rats at 72 hours and 7 days survival after axotomy (p<0.01, two-tailed *t*-test).

### Fractin Expression by dLGN Projection Neurons

Fractin-IR was seen only in regions of the brain with predictable cell death based upon a region's relation to the damaged visual cortex. For example, fractin-IR was observed in the ipsilateral cortex near the site of the lesion, and in a few of the dorsal-lateral thalamic nuclei that are known to project to the visual cortex. However, our analysis of the development of fractin-IR focused on neurons in the dLGN ipsilateral to the visual cortex lesion. It is important to note that rats studied for fractin-IR with a biotinylated secondary antibody and DAB did not receive cortical injections of BDA to avoid confounding BDA-labeling with fractin labeling. Nevertheless, it is reasonable to conclude that the observed fractin-labeled neurons in these rats are projection neurons as less than 1% of dLGN interneurons die or undergo apoptosis following a visual cortex lesion [22]–[25].

**Figure 14:**
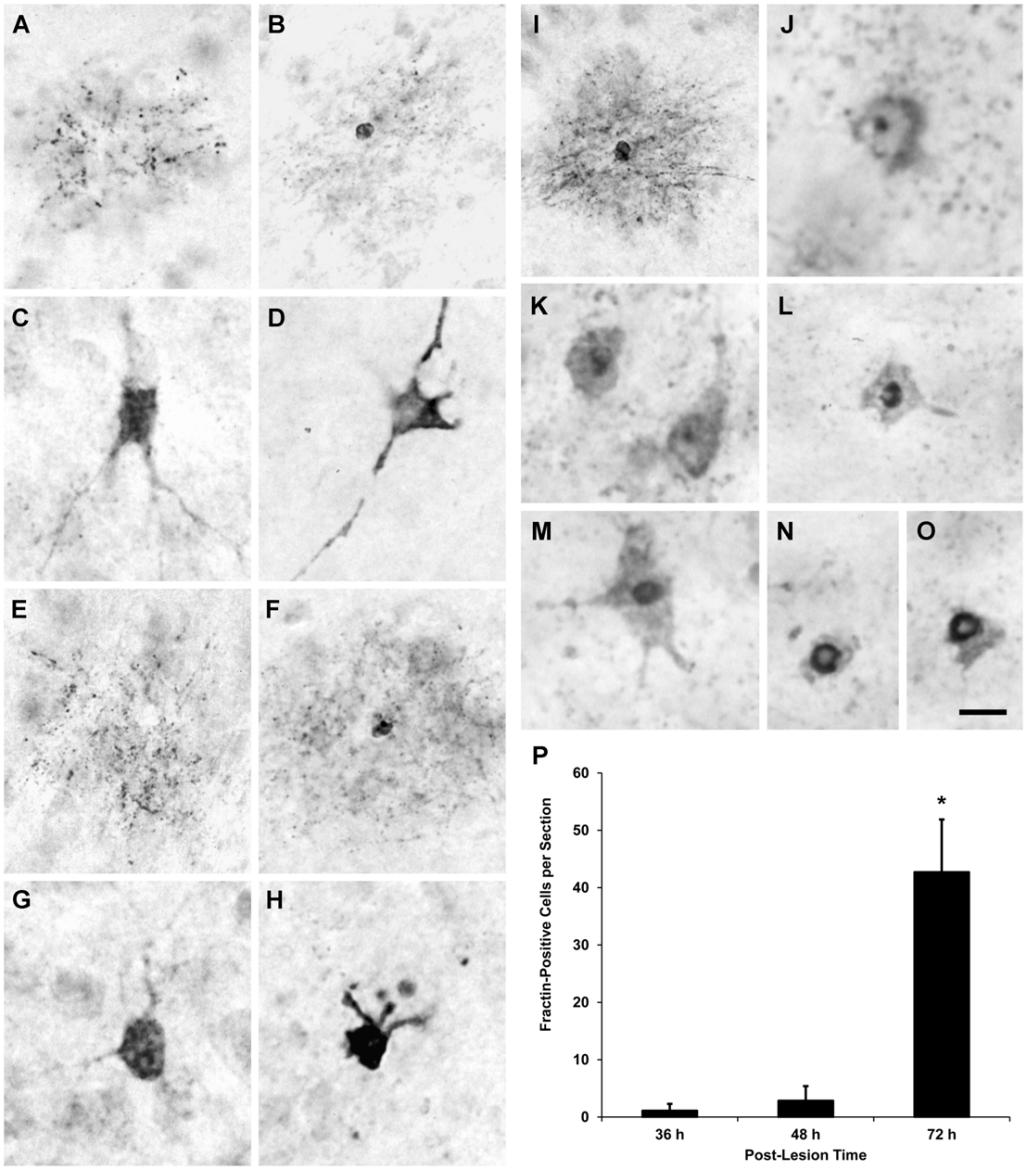
Fractin-IR neurons in the dLGN at (A–D) 36 hours, (E–H) 48 hours, (I, J) 72 hours, and (K–O) 7 days after a lesion of the visual cortex. (**A**) At 36 hours, dLGN neurons frequently displayed punctate fractin-IR in their dendrites, and (**B**) less often diffuse fractin-IR in the cell soma and punctate fractin-IR in the surrounding dendrites. (**C**, **D**) However, in some dLGN neurons, fractin-IR was evident in cell somas and primary dendrites. At 48 hours, fractin-IR dLGN neurons showed staining similar to that observed in neurons at 36 hours; namely, (**E**) Fractin-IR only as punctate staining in dendrites, (**F**) Fractin-IR in the cell soma and surrounding dendrites, or (**G**, **H**) dense fractin-IR in the cell soma and primary dendrites. (**I**) At 72 hours, fractin-IR in cell somas and surrounding dendrites was dense, and (**J**) in some dLGN neurons, fractin-IR was evident in the nucleolus. (**K**) At 7 days, dLGN neurons exhibited dense fractin-IR in the cell soma, while the cell somas of other fractin-IR cells contained (**L**, **M**) condensed nuclei and (**N**, **O**) disrupted membranes. (**P**) Few fractin-IR neurons were observed in the dLGN 36 hours after a lesion of the visual cortex. The number of fractin-IR neurons seen in the dLGN increased slightly in the next 12 hours, and then dramatically during the following 24 hours. The number of fractin-IR dLGN neurons at 72 hours after a lesion of the visual cortex was significantly different from the number at 36 or 48 hours, *p<0.001 (two-tailed *t*-test, n = 3 for each group). Scale bar: 30 µm (**A**, **B**, **E**, **F**), 60 µm (**C**, **D**, **G**, **H**), 50 µm (**I**), 25 µm (**J**), and 20 µm (**K**–**O**).

In rats that survived for 24 hours after a lesion of the visual cortex, fractin-IR was not seen in the dLGN ipsilateral to the cortical lesion, but at 36 hours survival, scattered fractin-IR in the ipsilateral dLGN was evident. This fractin-IR appeared mainly as loose clusters of fractin-positive puncta that appeared to be scattered along the dendrites of dLGN projection neurons (Figure 14A). Occasionally, in the center of the clusters, a cell soma was observed that was partially or completely filled by diffuse fractin-IR (Figure 14B). To a lesser extent, a few neurons also showed diffuse fractin-IR in both their cell somas and primary dendrites, but lacked punctate staining in their higher-order dendrites (Figures 14C, D). The clusters of fractin-IR puncta were distributed throughout the dLGN, and the density of the stained puncta varied significantly among the clusters. Each cluster represented a fractin-positive neuron that could be discriminated clearly because of the low density of the clusters. At 36 hours survival, only 1.2 fractin-IR cells, on average, were found in each dLGN section, representing approximately 0.1% of the total number of neurons in the dLGN [22].

The number of fractin-IR neurons increased slightly within the next 12 hours, resulting in the detection of 2.9 fractin-IR cells, on average, in each dLGN section. Although the number of fractin-IR neurons in the dLGN was greater at 48 hours survival than at 36 hours survival, the distribution and appearance of the fractin-IR neurons was similar at the two survival periods (Figures 14E–H). However, at 72 hours survival after the cortical lesion, fractin-IR in the dLGN had increased significantly. Fractin-IR in the dendrites and cell somas of dLGN neurons was pronounced (Figure 14I), and in some neurons, fractin-IR filled the cell soma cytoplasm densely (Figure 14J). The number of fractin-IR cells in the dLGN also increased rapidly between 48 and 72 hours survival, resulting in about a 15-fold increase in the total number of fractin-IR neurons in the dLGN at 72 hours survival compared with 48 hours survival (Figure 14P). These results suggest that the percentage of fractin-IR neurons had increased from 0.1% of all the cells in the dLGN at 36 hours survival after the cortical lesion to 4% of all the cells at 72 hours survival.

During the next four days, fractin-IR in the dLGN increased dramatically, and at 7 days survival dense fractin-IR puncta filled the entire dLGN ipsilateral to the cortical lesion. Because of the intense overlap of fractin-IR cells and processes, it was difficult at 7 days survival to distinguish individual fractin-IR neurons. Nevertheless, it was possible to discern that many dLGN neurons displayed fractin-IR in their nuclei (Figures 14K–M) and some fractin-positive neurons displayed disrupted cell soma membranes, indicating a late stage of cell death (Figures 14N,O).

**Figure 15:**
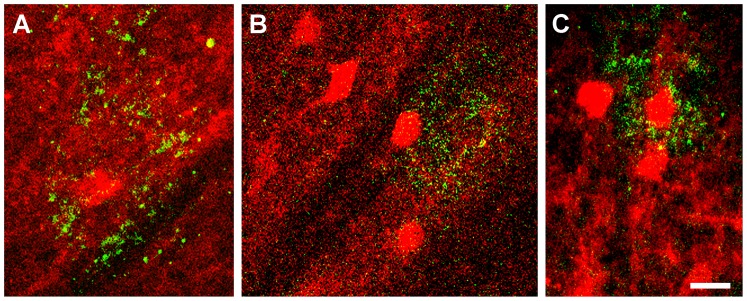
Fractin-IR projection neurons in the dLGN 72 hours after a lesion of the visual cortex that have been retrogradely labeled with Texas Red conjugated dextran (TRD). (**A**, **C**) TRD-labeled cell somas (red) surrounded by dendrites displaying fractin-IR puncta (green). (**B**) A TRD-labeled cell soma (red) associated with asymmetrically distributed fractin-IR dendrites (green). Note that nearby TRD-labeled cell somas are not accompanied by fractin-IR dendrites. Scale bar: 25 µm.

To confirm that axotomized dLGN projection neurons were fractin positive, we identified projection neurons that had been labeled retrogradely following injections of TRD into the visual cortex. FITC immunofluorescence was used to detect fractin-IR in sections through the dLGN. Neurons that had been labeled with TRD (red) and were positive for fractin (green) were recognized as fractin-IR dLGN projection neurons (Figures 15A–C).

These results demonstrate the involvement of activated caspase-3 in the death of axotomized projection neurons in the dLGN, and show that caspase-3 activity is evident as early as 36 hours after axotomy, and that it develops slowly and asynchronously in injured dLGN neurons during the next 12 hours. The expression of activated caspase-3 then increases dramatically in the following 24 hours and is observed in all of the cellular compartments of dLGN neurons that have been affected by a lesion of the ipsilateral visual cortex.

## Discussion

### Structural Changes in Axotomized dLGN Projection Neurons

In this study, we have used BDA as a retrograde tracer to demonstrate the structural changes that occur in the dendrites of confirmed dLGN projection neurons following axotomy during very early and later stages of cell death. In addition to confirming that cytological changes that take place in the cell somas of axotomized projection neurons in the dLGN at relatively late stages of injury, 3 days and thereafter, we demonstrated a series of structural changes in the dendrites of injured dLGN neurons as early as 12 hours after axotomy, well before changes in cell somas can be observed. This report also provides evidence that axotomized dLGN projection neurons in the rat can survive for at least 7 days after axotomy. Nevertheless, none of the observed surviving projection neurons appeared normal, which is perhaps to be expected as approximately 70% of the neurons in the dLGN ipsilateral to a cortical lesion have died at 7 days after a lesion of the visual cortex [22], [29]. The results further demonstrate that activated caspase-3 is involved in the structural and cytological changes that occur in axotomized projection neurons that precede their death. However, the present results demonstrate that a single administration of FGF2 at the site of a lesion of the visual cortex immediately after a lesion is made, is sufficient to retard the adverse structural and cytological consequences of axotomy that are displayed by dLGN projection neurons in untreated rats.

In order to rescue injured neurons from death and to reduce injury-induced functional deficits, it is necessary to understand the cytological and molecular changes that occur sequentially in neurons following an injury, particularly during the period before injured neurons commit to dying. Axotomized projection neurons in the dLGN proceed through an orderly series of events before dying, perhaps leaving open a “window of opportunity” during which time the death of some injured neurons may be averted by the administration of appropriate pharmacological agents or neuroprotective factors.

While injured neurons usually display various cytological changes, the best characterized changes have focused on cell soma atrophy, nuclear condensation and DNA fragmentation; all relatively late stage occurrences when cell death has become an irreversible fate choice of an injured neuron. By contrast, relatively little attention has been paid to the structural changes that occur in neurons soon after an injury is experienced. In adult rats, counts of neuronal cell somas show that approximately 99% of dLGN neurons ipsilateral to a lesion of the visual cortex remain intact during the first 72 hours after the lesion is made, but during the next four days 66% of the neurons in the dLGN die [22]. dLGN neurons axotomized by a lesion of visual cortex display cell soma atrophy, nuclear condensation, and DNA fragmentation, as noted, but these indices of cell injury only begin to become evident at three days after axotomy [22].

Dendritic changes have been reported by others in CNS neurons after various *in vivo* insults [1]–[9], [57]. Such changes often occur immediately following injury, but do not always lead to cell death. Under certain conditions dendritic alteration is a reversible event in injured neurons. Moreover, in intact animals, neurons such as axotomized rubrospinal projecting neurons, which demonstrate injury-induced dendritic alteration, can survive for over a month after axotomy [5], indicating that this type of injury does not lead inevitably to the rapid cell death of all CNS neurons.

This result raises the possibility that neurons that have experienced an injury that results in structural changes in their dendrites may retain the potential to survive if the early, injury-induced dendritic degeneration can be blocked from proceeding further. Initial changes in dendritic morphology resulting from injury, such as the formation of varicosities or beading, may not have a significant effect on cell survival, while a loss of secondary dendrites is likely to lead to cell death as secondary or higher-order dendrites support the majority of dendritic appendages that are critical for inter-neuronal communication. Therefore, when an injured neuron has lost its secondary or higher-order dendrites, it also loses its ability to communicate with other neurons, which results in neuronal dysfunction and often cell death. In this study, we observed that only those axotomized projection neurons that retained secondary dendrites, even if relatively few in number and short in length with respect to their normal counterparts, survived for at least seven days after axotomy. This observation suggests that maintaining a minimum number of secondary dendrites after axotomy may be a necessary requirement to extend neuronal survival and prevent rapid cell death.

As described here, dLGN projection neurons displayed an orderly sequence of dendritic alterations during the first 72 hours after axotomy before cytological changes in their cell somas were seen. As early as 12 hours after axotomy, the dendrites of injured dLGN projection neurons had begun to lose their appendages and distal segments of secondary dendrites. Within the next 48 hours, axotomized projection neurons gradually lost all of the distal segments of their dendrites. The sequence of dendritic degeneration, from the loss of appendages and the formation of varicosities to the disappearance of secondary dendrites, generally was consistent among cells in each of the three classes of projection neurons that we have described and from cell to cell within each class.

However, the initiation, duration and endpoint of the sequence varied among axotomized dLGN projection neurons. Thus while all dLGN projection neurons in this study experienced a comparable injury and displayed similar structural alterations in their dendrites soon after axotomy, the extent of these alterations varied with the class of projection neuron, and often differed among neurons within each class of projection neurons, regardless of the survival time after axotomy. Among the three classes of projection neurons, cells that retained four or more primary dendrites after axotomy were radial or basket cells. In contrast, bipolar cells experienced severe dendritic degeneration and disappeared from the dLGN earlier than basket or radial cells. These results suggest that the three classes of dLGN projection neurons differ in their vulnerability to axotomy, bipolar cells being more vulnerable than basket cells which in turn are more vulnerable than radial cells. The reasons for this differential vulnerability remain to be determined, but it may be related to the number of afferent synapses that each class of projection neuron can support after axotomy. The number of dendrites, and, therefore, the opportunity for synaptic connections is greater in radial cells than in basket cells and least in bipolar cells [33].

In summary, dendritic degeneration is a very early event in adult rat dLGN neurons after axotomy. Changes in the dendritic structure of dLGN projection neurons emerge within 12 hours after axotomy. These dendritic changes continue during the next 48 hours, during which time most dLGN projection neurons lose their secondary dendrites. The disappearance of secondary dendrites is accompanied by cell soma atrophy, which is underway 72 hours after axotomy. Since dLGN projection neurons undergo a predictable sequence of structural alterations after axotomy, these alterations may be used as criteria to discriminate among different degenerative stages of injured neurons, and may be useful as reference points to investigate the biochemical and molecular events that underlie cell death after axotomy at defined stages.

### Caspase-3 Activity in Axotomized dLGN Projection Neurons

In this report we also have confirmed caspase-3 activity in axotomized dLGN projection neurons using an antibody raised against an actin fragment, fractin, which results solely from the cleavage of beta actin by activated caspase-3. While calpain may cleave actin in addition to caspase-3, fractin is a specific 32 kD product produced solely by the cleavage of actin by caspase 3 as reported by several previous studies that involved: (a) the immunolabeling of Hirano bodies, which are eosinophilic rod-shaped inclusions found primarily in CA1 hippocampal neurons in aged animals and in the brains of Alzheimer's disease patients [53]; (b) a demonstration that the caspase-specific cleavage product fractin is produced in oligodendroglial cells during TGF-beta-mediated apoptosis [54]; (c) distinguishing between calpain and caspase-3 activity by staining for fractin in excitoxicity-induced neurodegeneration in adult brains [55] and in Alzheimer neuropathology [56], [58]. During apoptosis, beta actin is cleaved by activated caspase 3 between Asp244 and Gly245. This cleavage results in the release of an approximately 130 amino acid (15 kDa) fragment from the C-terminus of beta actin and a 32 kDa (244 amino acid) N-terminal fragment of actin known as fractin. The antibody that we used was raised against fractin, and positive staining, therefore, is evidence that actin has been cleaved by caspase 3. Thus, while calpain may be capable of cleaving actin, this capability is not relevant to the immunostaining that was employed, because calpain cleavage of actin would not generate fractin, and, therefore, our interpretation that the immunostaining results indicate the involvement of caspase-3 is correct.

However, it is important to note that the caspase-3 activity that has been detected in these experiments may underestimate its activity, because beta actin is only one of several substrates of caspase-3. Moreover, caspase-3 may be activated earlier than reported here to cleave other substrates, such as α-spectrin and gelsolin, which have not been examined in this study. Nevertheless, the present results demonstrate clearly that caspase-3 is active in axotomized dLGN neurons, and they illustrate the temporal and spatial pattern of this activity in projection neurons in the dLGN ipsilateral to a lesion of the visual cortex.

Axotomized dLGN neurons have been thought to die apoptotically, because they display morphological characteristics typically associated with apoptosis [22]–[25]. The present results showing the involvement of caspase-3 in the death of axotomized dLGN projection neurons adds additional support to the notion that these cells undergo apoptosis after axotomy. Caspase-3 activity in the dLGN ipsilateral to a lesion of visual cortex was first detected at 36 hours survival. While this is 24 hours later than the first observed degeneration in the dendrites of axotomized dLGN projection neurons, it precedes by about 36 hours the initial appearance of injury-induced cytological changes in the cell soma of these neurons [22]. This result suggests that the earliest dendritic changes, which are observed 12 hours after the axotomy of dLGN projection neurons, are caused by the cleavage of proteins other than beta actin, either by caspase-3 or by other proteases such as calpain [59], functioning alone or in concert with caspase-3 [60]–[65].

That caspase-3 also may be activated later than other proteases in axotomized dLGN projection neurons is consistent with the observation in the present study that the appearance of intense fractin-IR in the dLGN occurs first at 72 hours after axotomy, well after dendritic degeneration is initially detected, and is followed soon thereafter by a period of massive cell death in the dLGN [22]. Levels of activated caspase-3 have been reported to peak 5 to 6 days following a cortical lesion, coincident with the expression of p53 [30], [31], and are preceded by indicators of oxidative stress [31], [66]. This sequence of events is consistent with the intense fractin-IR observed 7 days following the cortical lesion in these experiments. Furthermore, recent studies have shown that the inhibition of caspase-3 activity only delays cell death after injury, but does not prevent it from occurring. Even when caspase-3 activity is inhibited, injured cells continue to die, but they do so by necrosis instead of apoptosis [67]–[70]. The results from these studies and those presented here suggest that caspase-3 activity is most intense at relatively late stages of cell injury, when cell death has become irreversible. Therefore, it is likely that fulminant caspase-3 activity signals the irreversible death of an injured neuron.

Activated caspase-3 also is implicated in the death of axotomized dLGN neurons in young rats and mice. In rats, removal of the visual cortex during the first two postnatal weeks results in the rapid, massive death of neurons in the dLGN with complete loss of the nucleus by adulthood [71]. TUNEL- and activated caspase-3-positive cells were seen in the dLGN 24 to 72 hours following a lesion of the visual cortex, but were rarely observed 7 days after the lesion [71]–[73]. These results indicate that caspase-3 activity accompanying axotomy-induced cell death in the dLGN of young rats and mice is initiated rapidly after axotomy and peaks in intensity shortly thereafter.

In contrast, in axotomized dLGN projection neurons in the adult rat fractin-IR was detected first in dendrites, the most vulnerable compartment of these neurons at 36 hours survival. Only after the intensity of fractin-IR had increased in both dendrites and cell somas at 72 hours survival was fractin-IR detected in cell nuclei, which often appeared condensed. It is perhaps not a coincidence that the dendrites of axotomized dLGN projection neurons degenerate progressively during the first three days after axotomy. At 72 hours survival, approximately 60% of the dendrites of axotomized dLGN projection neurons have been lost, and cell somas have begun to atrophy and display nuclear condensation. These results indicate that the temporal progression of caspase-3 activity in axotomized dLGN projection neurons follows with a delay the same sequence of structural changes that are observed in these neurons; specifically, the structural changes seen in axotomized dLGN projection neurons and caspase-3 activity appear first in the dendrites of the injured neurons, and then in the next two days advance to involve the cell soma, preceding the death of the injured neuron. In an axotomized dLGN projection neuron, the progression of cytoskeletal degeneration and caspase-3 activity from dendrites to the cell soma is a roadmap leading to the death of the injured neuron. Interrupting this roadmap may offer an opportunity to protect axotomized neurons from dying.

With this in mind, we investigated interrupting the roadmap with a single administration of FGF2 at the site of the cortical lesion to determine qualitatively and quantitatively if this affected the time course and extent of the dendritic degeneration in axotomized projection neurons in the rat dLGN. The present results clearly demonstrate that a single administration of FGF2 significantly reduces, but does not prevent, the degeneration of dendrites in these axotomized neurons.

As mentioned, when the dendrites of injured dLGN projection neurons degenerate, the ability of these neurons to receive information from other cells and participate functionally in neuronal circuits is compromised. When axotomized projection neurons in the dLGN of the rat lose more than 50% of their dendrites, they frequently die. By contrast, when dLGN projection neurons retain more than 10 dendrites and a dendritic arbor with a cross-sectional area at least 20% of normal size, some projection neurons survive up to 7 days after axotomy. Thus, maintaining a minimum number of dendrites and a dendritic arbor of minimal dimensions appears to be associated with the survival of dLGN projection neurons after axotomy. Therefore, a reduction in dendritic degeneration following axotomy may play an important role in protecting injured dLGN projection neurons from dying.

Administration of FGF2 *in vivo* has been shown to reduce neuronal death in the adult brain triggered by axotomy [74]–[80], excitotoxicity [81]–[83], MPTP treatment [84]–[87], and traumatic injury [88]. We have shown here that a single administration of FGF2 at the time of axotomy reduces the number of dendrites of dLGN projection neurons that otherwise would die, promotes the retention of appendages by these spared dendrites, and moderates the decrease in the size of projection neuron dendritic arbors seen in untreated rats. Since the same administration of FGF2 has been demonstrated to markedly increase the survival of axotomized dLGN neurons, while not preventing cell soma atrophy [40], it is reasonable to suggest that the reduction in early dendritic degeneration by FGF2 reported here plays an important role in the survival of axotomized dLGN projection neurons.

Of interest is that the effect of FGF2 treatment on reducing the loss of dendrites in axotomized dLGN projection neurons was observed for all three classes of projection neurons. In normal rats bipolar cells are the most vulnerable dLGN projection neurons to axotomy, nearly disappearing as a cell class of projection neurons at 7 days. By contrast, radial cells are the most resistant projection neurons, and many of them survive in the dLGN 7 days after axotomy. However, in FGF2-treated rats, all three classes of dLGN projection neurons are seen 7 days after axotomy, and, notably, some bipolar cells with normal appearing dendritic trees are present at 7 days (Figure 10).

It is important to note, however, that the protection afforded by a single treatment with FGF2 does not affect all axotomized dLGN projection neurons uniformly. While neurons from each of the three classes of dLGN projection neurons, radial, basket and bipolar cells, often appeared nearly normal in FGF2 treated rats 7 days after axotomy (Figure 10), other radial, basket and bipolar projection neurons displayed clear dendritic degeneration in FGF2 treated animals at this survival time (Figure 12). The reason for this discrepancy is not clear, but may be related to the FGF2 dose that was used and/or the short treatment duration afforded by a single administration of FGF2. Extending the FGF2 treatment over several consecutive days after axotomy, or increasing the dose of FGF2 used might result in more uniform neuroprotection of axotomized projection neurons.

Although the neuroprotective effects of FGF2 after axotomy have been widely acknowledged, as mentioned, the intracellular signaling mechanisms involved remain unclear. In cultured rat cortical neurons, FGF2 treatment increased ERK1/2 phosphorylation resulting in prolonged neuronal survival [89]. In the frog visual system, FGF2 applied to the optic nerve of axotomized retinal ganglion cells prolonged the activation of extracellular signal-related kinase (ERK), increased Bcl-2 and Bcl-x_L_ expression while decreasing the expression of the pro-apoptotic protein Bax, and blocked the cleavage of caspase 3 [90]. In the rat visual system, retinal ganglion cell axotomy has been observed to result in the activation of mitogen-activated protein kinase (MAPK), and the phosphorylation of cAMP-responsive element (CREB), ERK1/2 and Akt [91], [92]. If one or more of these signaling molecules also is activated when geniculocortical axons are axotomized, then it is not unreasonable to speculate that the neuroprotective effects of FGF2 treatment may result from the interaction of FGF2 with one or more of these molecules leading to the upregulation of pro-survival proteins, such as Bcl-2 and/or Bcl-x_L_, the down regulation of pro-death proteins, such as Bax, PARP or DFF, and the blockage of caspase-3 cleavage.

In summary, the degenerative changes in the dendrites of dLGN projection neurons can be observed as early as 12 hours after axotomy, and are among the most rapidly occurring consequences of cell injury. That FGF2 retards the degeneration of the dendritic cytoskeleton and increases the survival of dLGN projection neurons after axotomy strongly suggests that FGF2 may inhibit proteases such as calpain and caspase-3 from cleaving the cytoskeletal proteins that are essential to dendritic integrity, as well as interfering with the cleavage of pro-apoptotic proteins that are directly involved in initiating apoptotic cell death. Since cytoskeletal protein degradation is the direct cause of the axotomy-induced changes in cell structure that ultimately may lead to cell death, it is reasonable to suggest that the efficacy of FGF2 as a neuroprotective factor is due in part because it may function as an inhibitor of cytoskeletal or pro-apoptotic proteases upon binding to an injured neuron.
